# Structural robustness of networks with degree-degree correlations between second-nearest neighbors

**DOI:** 10.1371/journal.pone.0336970

**Published:** 2025-12-05

**Authors:** Yuka Fujiki, Stefan Junk

**Affiliations:** 1 Frontier Research Institute for Interdisciplinary Sciences (FRIS), Tohoku University, 6-3 Aramaki aza Aoba, Aoba-ku, Sendai, Japan; 2 Advanced Institute for Materials Research (AIMR), Tohoku University, 2-1-1 Katahira, Aoba-ku, Sendai, Japan; 3 Gakushuin University, 1-5-1 Mejiro, Toshima-ku, Tokyo, Japan; Beijing University of Technology, CHINA

## Abstract

We numerically investigate the robustness of networks with degree-degree correlations between nodes separated by distance *l* = 2 in terms of shortest path length. The degree-degree correlation between the *l*-th nearest neighbors can be quantified by Pearson’s correlation coefficient *r*_*l*_ for the degrees of two nodes at distance *l*. We introduce *l*-th nearest-neighbor correlated random networks (*l*-NNCRNs) that are degree-degree correlated at less than or equal to the *l*-th nearest neighbor scale and maximally random at farther scales. We generate 2-NNCRNs with various *r*_1_ and *r*_2_ using two steps of random edge rewiring based on the Metropolis-Hastings algorithm and compare their robustness against failures of nodes and edges. As typical cases of homogeneous and heterogeneous degree distributions, we adopted Poisson and power law distributions. Our results show that the range of *r*_2_ differs depending on the degree distribution and the value of *r*_1_. Moreover, comparing 2-NNCRNs sharing the same degree distribution and *r*_1_, we demonstrate that a higher *r*_2_ makes a network more robust against random node/edge failures as well as degree-based targeted attacks. This behavior was observed in nearly all simulated cases, except for highly assortative power-law networks, where the relationship is more complex.

## 1 Introduction

Many complex networks in the real world share a common property that the distribution of the number of edges (degree) from each node is approximated by a power-law function, which is known as the scale-free property [1]. The degree correlation among nodes in such networks, particularly nearest-neighbor degree correlation (NNDC), influences network behavior, including robustness to node and edge failures, the spread of infections, and oscillator synchronization [2–4].

**Fig 1:**
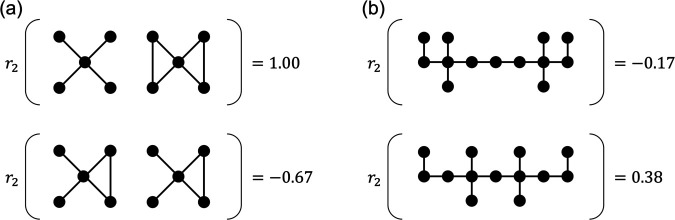
Examples of small networks with identical NNDC but different LRDCs. (a) Low-degree nodes are clustered around high-degree hubs, but the average degree of neighbors differs between hubs. This asymmetry leads to different *r*_2_’s despite *r*_1_ being fixed, where NNDC is quantified by *r*_1_ and LRDC at distance *l* = 2 is quantified by *r*_2_, both defined in Eq (1). (b) In the first row, hubs are not directly connected and are separated by a relatively long distance, suppressing indirect high-degree interactions and leading to a negative *r*_2_.

Additionally, recent studies have shown that the majority of complex networks in the real world exhibit long-range degree correlations (LRDCs) at greater distances than next-nearest neighbors [5]. For example, airlines often use a “hub-and-spoke" model and protein-interaction networks are structured into functional modules consisting of a high-degree “hub” protein and low-degree peripheral proteins[6]. They have typically negative NNDCs, yet can exhibit diverse second-neighbor structures depending on how hubs are surrounded by and connected via low-degree nodes. To illustrate such mechanisms, Fig 1 presents schematic examples where networks with identical NNDCs differ in LRDCs at distance *l* = 2 due to different global arrangements.

While some LRDCs can be explained as “extrinsic" LRDCs resulting from the propagation of the NNDCs, for the most part, these are “intrinsic" LRDCs that are not merely consequences of the NNDCs [7]. The effects of LRDCs at distance *l*>1 remain poorly understood, except for some special cases. For example, it is known that the shortest path length between high-degree nodes (hub) influences network functions and dynamical properties. Greater hub separation can reduce traffic congestion in transport networks [8], suppress pandemic onset in epidemic networks [9], and shape information flow in brain networks [10]. Repulsive correlation between hubs is also closely related to fractal structure [11,12], which is associated with anomalously slow diffusion and a fragile nature under node removal. Furthermore, transsortative structures (degree correlations between two neighbors of a node) can amplify the majority illusion [13]. However, a comprehensive understanding of their effects on network structure and dynamics, even for the simplest case *l* = 2, is still lacking. Towards understanding the general relationship between network properties and LRDCs, in this study, we numerically investigate the influence of LRDC at *l* = 2 on the structural robustness, which is one of the most fundamental properties of networks.

For the purpose of this work, robustness refers to the ability of a network to maintain its overall structural integrity against node or edge removal [14–18]. Positive (negative) NNDCs are known to make the network more robust (weaker) and tend to delay (advance) the loss of global connectivity, i.e., global connectivity is lost after a larger (smaller) percentage of random failures or targeted attacks [19–22]. This robustness concept can be interpreted as assessing the stability of network functioning, or the difficulty of eradicating infectious diseases and misinformation spreading on the network. For instance, robustness reflects the ability to deliver messages despite failures in communication networks. Also, in epidemic processes, node and edge removal can represent vaccination and the suppression of transmission pathways, respectively. Understanding the effect of LRDCs on the network robustness provides valuable insights into the resilience of networks and should lead to more effective system design.

As the framework for this investigation, we introduce *l*-th nearest-neighbor correlated random networks (*l*-NNCRNs) that are degree–degree correlated up to the *l*-th nearest-neighbor distance and maximally random at any further scale. Formally, LRDCs at distance *l* are characterized by the conditional probability $P(k,k^{\prime }|l)$ that the degrees of two nodes are *k* and $k^{\prime }$ given that the nodes are separated by *l* steps. When we compare properties of *l*-NNCRNs with the same correlations $P(k,k^{\prime }|l^{\prime })$ at distances $l^{\prime }\leq l\;-\;1$, any differences in their properties can be attributed to intrinsic LRDCs at distance *l*, making this framework a convenient tool for isolating their effects.

We numerically implement this framework in the case *l* = 2 for a representative choice of correlations, the strength of which is quantified and controlled by Pearson’s correlation coefficient *r*_*l*_ for $P(k,k^{\prime }|l)$. We generate 2-NNCRNs with various *r*_1_ and *r*_2_ using two steps of random edge rewiring based on the Metropolis-Hastings algorithm. Our results demonstrate, firstly, that the correlations at distances *l* = 0 and *l* = 1, i.e. the degree distribution and *r*_1_, place some constraints on the range of *r*_2_. We then perform random node or edge removal and degree-based targeted attack on networks and quantify the robustness based on the percolation critical point *f*_*c*_ and the robustness measure *R* introduced by Schneider *et al.* [23]. Both measures assess slightly different aspects of robustness, as will be explained in Sect 3.2.

Qualitatively, our results suggest that both *f*_*c*_ and *R* shift with *r*_2_ even when *r*_1_ is fixed, demonstrating that LRDCs influence network robustness. Somewhat surprisingly, the effect of *r*_2_ on robustness follows a similar trend to that of *r*_1_. The influence of *r*_2_ on network robustness is weaker than that of *r*_1_, but it is generally on a comparable order. Networks with larger *r*_2_ tend to be more robust against both random node/edge failure and degree-based targeted attacks when the robustness is quantified by *f*_*c*_. Using another measure of robustness, *R*, networks with larger *r*_2_ are more fragile against random node/edge failure but remain more robust against degree-based targeted attacks.

To clarify how degree correlations at distance *l* = 2 affect network robustness, we decompose 2-NNCRNs into *k*-cores and confirm that a strongly positive *r*_2_ induces a core, which is as robust as the core induced by *r*_1_. This *k*-core perspective, together with the superedge interpretation given at the end of Sect 4, explains the similarity in the effects of *r*_1_ and *r*_2_ observed in our results. Finally, we examine the values of *r*_2_ exhibited by real networks and the possibility for optimization of robustness based on LRDCs. Beyond the structural robustness, a complete picture of real-world dynamics requires addressing further aspects, such as the efficiency of information flow or robustness against cascading failures. We briefly comment on these concepts in Sect 5.2. We also outline potential extensions of our framework to higher-order correlations (l≥3) and to more complex failure scenarios.

The manuscript is organized as follows. In Sect 2 we define *l*-NNCRNs by quantifying LRDCs using $P(k,k^{\prime }|l)$ and *r*_*l*_. Moreover, we explain the algorithm for generating 2-NNCRNs with various values of *r*_*l*_ using random edge rewiring based on the Metropolis-Hasting algorithm. Sect 3 presents the numerical results obtained from our simulations, including the effects of LRDCs on network robustness and the relationship between *r*_1_, *r*_2_ and the degree distribution. Finally, in Sect 4 we summarize our key findings and in Sect 5 propose avenues for future research in elucidating the role of LRDCs in complex networks.

## 2 Numerical method

We numerically sample networks with various degree-degree correlations between nodes at distance l≤2 in terms of shortest path length.

We propose *l*-th nearest neighbor correlated networks (*l*-NNCRNs), which are random at shortest path distances greater than *l* and which serve as an idealized model to study the relation between network robustness and LRDCs without other confounding factors. 2-NNCRNs are sampled using a three-step edge-rewiring algorithm to fix, successively, the degree sequence, the NNDCs and finally the LRDCs at distance *l* = 2. The definition and sampling method of *l*-NNCRNs are explained in the following two sections.

Note that in real-world networks, the effect of *r*_2_ is complicated by the fact that *r*_1_ by itself also influences network robustness and, on the other hand, constrains the range of *r*_2_. In addition, robustness may be affected by intrinsic long-range correlations at distances l≥3 or other complex structural features, such as multi-scale or non-Markovian dynamics. The 2-NNCRN is an idealized model designed to disentangle the specific effect of second-neighbor correlations from these confounding factors. By generating networks with an identical degree distribution and *r*_1_ while systematically varying *r*_2_, we isolate the effect of LRDCs at distance *l* = 2 and demonstrate that they have a profound effect on network robustness. To investigate the relation between the robustness and LRDCs at *l* = 2, we compare the effect of random node/edge removal and of degree-based targeted attack on the network structure in 2-NNCRNs with different degree correlations. The analysis of these properties is explained in the final section.

### 2.1 Definition of *l*-NNCRNs

To analyze LRDCs in a given network, we first need to understand maximally random networks that have the same nearest-neighbor correlations as the network [7]. We can use that model as a baseline to judge whether the degree correlations at greater distances than *l* = 1 are just extrinsically caused by the NNDCs or whether they are intrinsic. Extending this, we call a network ensemble *l*-NNCRN if it is degree-degree correlated at less than or equal to the *l*-th nearest neighbor distance and maximally random at any further scale. A nearest-neighbor correlated random network is thus a 1-NNCRN.

The conditional probability $P(k,k^{\prime }|l)$ that two randomly chosen nodes separated at a distance *l* have degree *k* and $k^{\prime }$ describes the LRDCs at distance *l*. Since the conditional probability is a high-dimensional matrix and not easy to interpret, we use the Pearson’s correlation coefficient *r*_*l*_ for the degrees of two nodes at distance *l* as a convenient observable that quantifies the strength of the *l*-th nearest neighbor degree correlation. It is defined as

$$r_{l}=\frac{\sum_{k,k^{\prime }}kk^{\prime }P(k,k^{\prime }|l)-[\sum_{k,k^{\prime }}kP(k,k^{\prime }|l){]}^{2}}{\sum_{k,k^{\prime }}k^{2}P(k,k^{\prime }|l)-[\sum_{k,k^{\prime }}kP(k,k^{\prime }|l){]}^{2}}\in [-1,1],$$ (1)

where the sum is over all possible degrees. It can also be represented as

$$r_{l}=\frac{\frac{1}{{2M}^{\left(l\right)}}\sum_{i,j}\delta_{\mathrm{dist}(i,j),l}\;k_{i}k_{j}-{\left[\frac{1}{{2M}^{\left(l\right)}}\sum_{i,j}\delta_{\mathrm{dist}(i,j),l}\;k_{i}\right]}^{2}}{\frac{1}{{2M}^{\left(l\right)}}\sum_{i,j}\delta_{\mathrm{dist}(i,j),l}\;k_{i}^{2}-{\left[\frac{1}{{2M}^{\left(l\right)}}\sum_{i,j}\delta_{\mathrm{dist}(i,j),l}\;k_{i}\right]}^{2}}.$$ (2)

Here, the sum is over all nodes, dist(i,j) is the shortest path distance between *i* and *j*. The term *M*${\;}^{\left(\text{l}\right)}$ is defined as

$$M^{\left(l\right)}=\frac{1}{2}\sum_{i,j}\delta_{\mathrm{dist}(i,j),l},$$ (3)

where $\delta_{d,l}$ is Kronecker *δ*. In the case of *l* = 1, this quantity is known as assortativity [2] and it has been extended to arbitrary *l*>1 by [24,25].

Note that the NNDCs can induce extrinsic LRDCs at distance *l* = 2, so that the value of *r*_2_ may differ from zero even if the network is a 1-NNCRN. For a given network, we thus define $r_{2}^{\mathrm{ext}}$ to be the average value of *r*_2_ among networks with the same degree sequence and NNDC, as described by $P(k,k^{\prime }|l=1)$. We say that a network is intrinsically degree-degree correlated at *l* = 2 if the value of *r*_2_ significantly differs from $r_{2}^{\mathrm{ext}}$.

Several other network ensembles can also exhibit long-range degree correlations (LRDCs), such as stochastic block models, hierarchical modular networks [26], and network-of-networks frameworks [27]. These models generate LRDCs through distinct mechanisms, including community structure, hierarchical organization, and intermodular connectivity. While illuminating, their robustness properties often require model-specific analysis and are not directly comparable with the maximally random *l*-NNCRNs used in this study. A detailed comparison with these alternative ensembles, as well as with motif-based correlation measures like the dk-series [28], is provided in Supporting Information.

### 2.2 Edge rewiring algorithm

Edge rewiring algorithms are widely used to investigate graph structures [28–30]. Through randomization via rewiring, the algorithm can generate the most randomized network ensembles under specific constraints. In this study, given parameters *J*_1_ and *J*_2_, we generate 2-NNCRNs through the following sequence of steps based on the Metropolis-Hasting algorithm started with an initial network *G*_0_: (1) Rewire edges while preserving the degree sequence to generate a 1-NNCRN *G*_1_(see Algorithm 1). (2) Rewire edges in the resulting network while preserving the NNDC (i.e., $P(k,k^{\prime }|l=1)$) to generate a 2-NNCRN *G*_2_ (see Algorithm 2).

Note that when choosing edges, we (temporarily) equip each edge with an orientation. We also mention that the rewiring in Algorithm 1 is irreducible for the set of graphs with the same degree sequence and that in Algorithm 2 is as well for the NNDC [31,32]. Moreover, the reason why we include Step 2 in both algorithms it to ensure that the Markov chains are aperiodic and thus converge to equilibrium.

**Algorithm 1 Generating a 1-NNCRN while preserving the degree sequence.**

1: Increase time step by 1.

2: Return to step 1 with probability 1/2.

3: Randomly choose two edges $\left(u_{1},u_{2}\right)$ and $\left(v_{1},v_{2}\right)$.

4: If rewiring $\left(u_{1},u_{2})\to (u_{1},v_{2}\right)$ and $\left(v_{1},v_{2})\to (v_{1},u_{2}\right)$ creates a loop or a multi-edge, return to step 1.

5: Compute the current value $r_{1}^{\text{prev}}$ of *r*_1_, the value $r_{1}^{\text{next}}$ of *r*_1_ after rewiring $\left(u_{1},u_{2})\to (u_{1},v_{2}\right)$ and $\left(v_{1},v_{2})\to (v_{1},u_{2}\right)$, and the transition probability $P(u_{1},u_{2},v_{1},v_{2})=\min(1,\exp[-J_{1}(r_{1}^{\text{next}}$ $-r_{1}^{\text{prev}})])$.

6: With probability $P(u_{1},u_{2},v_{1},v_{2})$, rewire edges $\left(u_{1},u_{2})\to (u_{1},v_{2}\right)$ and $\left(v_{1},v_{2})\to (v_{1},u_{2}\right)$.

7: Repeat steps 1–6 until the system reaches equilibrium.

**Algorithm 2 Generating a 2-NNCRN while preserving the degree sequence and $P(k,k^{\prime }|l=1)$.**

1: Increase time step by 1.

2: Return to step 1 with probability 1/2.

3: Randomly choose an edge (*u*_1_, *u*_2_).

4: Randomly choose another edge ($v_{1}$, $v_{2}$) such that $v_{1}$ has the same degree as *u*_1_.

5: If rewiring $\left(u_{1},u_{2})\to (u_{1},v_{2}\right)$ and $\left(v_{1},v_{2})\to (v_{1},u_{2}\right)$ creates a loop or a multi-edge, return to step 1.

6: Compute the current value $r_{2}^{\text{prev}}$ of *r*_2_, the value $r_{2}^{\text{next}}$ of *r*_2_ after rewiring $\left(u_{1},u_{2})\to (u_{1},v_{2}\right)$ and $\left(v_{1},v_{2})\to (v_{1},u_{2}\right)$, and the transition probability $P(u_{1},u_{2},v_{1},v_{2})=\min(1,\exp[-J_{2}(r_{2}^{\text{next}}$ $-r_{2}^{\text{prev}})])$.

7: With probability $P(u_{1},u_{2},v_{1},v_{2})$, rewire edges $\left(u_{1},u_{2})\to (u_{1},v_{2}\right)$ and $\left(v_{1},v_{2})\to (v_{1},u_{2}\right)$.

8: Repeat 1–7 until the system reaches equilibrium.

These algorithms generate a canonical ensemble of networks in the sense of statistical physics, which satisfies a constraint in terms of the mean value (soft constraint) and belongs to the general class of exponential random graph models [33–35].

More precisely, in Algorithm 1 the degree of each node does not change, so the degree sequence acts as a hard constraint. On the other hand, the rewiring generates a set of networks with different NNDCs but which is random at further distances, i.e., a 1-NNCRNs. The mean value of *r*_1_ is a soft constraint in Algorithm 1 and in equilibrium a configuration *G*_1_ (with the same degree sequence as the initial network *G*_0_) appears with probability

$$\Pi_{1}(G_{1}|G_{0})=\frac{e^{-J_{1}r_{1}(G_{1})}}{Z_{1}(J_{1}|G_{0})},$$ (4)

where *r*_1_(*G*) is the value of *r*_1_ for *G* and *Z*_1_ is the partition function

$$Z_{1}(J_{1}|G_{0})=\sum_{G^{\prime }}e^{-J_{1}r_{1}(G^{\prime })}.$$ (5)

**Fig 2:**
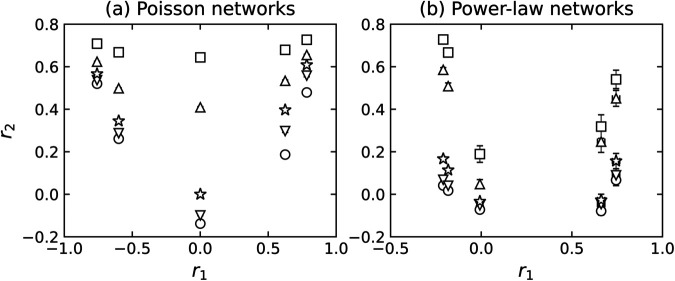
The mean value of *r*_1_ and *r*_2_ of 2-NNCRNs. Each data point is the average value over 100 configurations, and the error bar is the standard deviation. For *r*_1_, the error bars are smaller than the symbol size in all cases and are therefore not visible in the plot. The *r*_1_ and *r*_2_ of power-law networks exhibit larger error bars than those of Poisson networks, which can be attributed to significant differences in maximum degrees across random seeds. The symbols vary based on the rewiring parameter *J*_2_. For *J*_2_ = −2*M*,−*M*,0,*M*,2*M*, the symbols are a sphere, a downward triangle, a star, an upward triangle, and a box, respectively, where *M* is the number of edges in each network. In particular, the star indicates *J*_2_ = 0, where the value of $r_{2}^{\mathrm{ext}}$ reflects the extrinsic LRDC at *l* = 2 induced by *r*_1_ in 1-NNCRNs. If *r*_2_ is greater than/less than $r_{2}^{\mathrm{ext}}$, it implies that an intrinsic positive/negative LRDC is present at *l* = 2.

The mean value of *r*_1_ is zero in the case *J*_1_ = 0 and moves in the positive/negative direction with *J*_1_. In statistical physics, these parameters are analogous to an inverse temperature. As they are tuning parameters without a direct interpretation themselves, our analysis focuses on the resulting observable correlation coefficients, *r*_1_ and *r*_2_. Empirically, we found that these parameters should be chosen to be proportional to the number of edges in the network for the rewiring to be effective. Moreover, when the network size is sufficiently large, the value of *r*_1_ tends to be narrowly distributed around its mean value (notice that the error bars in Fig 2 are barely visible).

The idea behind Algorithm 2 is similar, but in addition to the degree sequence the condition that *u*_1_ and $v_{1}$ have the same degree ensures that $P(k,k^{\prime }|l=1)$ is preserved, and in particular that *r*_1_ remains constant. When the system reaches equilibrium in Algorithm 2, a configuration *G*_2_ with the same degree sequence and NNDC as *G*_1_ appears with probability

$$\Pi_{2}(G_{2}|G_{1})=\frac{e^{-J_{2}r_{2}(G_{2})}}{Z_{2}(J_{2}|G_{1})},$$ (6)

where *r*_2_(*G*) is the value of *r*_2_ for *G* and $Z_{2}(J_{2}|G_{1})$ is the partition function in the form of

$$Z_{2}(J_{2}|G_{1})=\sum_{G^{\prime }}e^{-J_{2}r_{2}(G^{\prime })}.$$ (7)

The mean value of *r*_2_ equals $r_{2}^{\mathrm{ext}}$ in the case *J*_2_ = 0 and moves in the positive/negative direction in accordance with *J*_2_. Both rewiring procedures satisfy the detailed balance condition. Thus, after successively applying Algorithms 1 and 2 to an initial network *G*_0_ until equilibrium is reached, we obtain network *G*_2_ with probability

$$\sum_{G_{1}}\Pi_{1}(G_{1}|G_{0})\Pi_{2}(G_{2}|G_{1}).$$ (8)

We prepare two kinds of random networks as the initial network *G*_0_, which prescribes the degree distribution of the network and which is preserved by Algorithms 1 and 2. The first is the Erdős-Rényi random graph model, whose degree distribution is approximated by a Poisson distribution $P(k)=\langle k\rangle^{k}e^{-k}/k!$ when the network is large enough. We set the number of nodes and the average degree at N=20,000 and ⟨k⟩=5.0, respectively. The second is the configuration model [36] with a power-law degree distribution $P(k)=ck^{-\gamma }$ with the number of nodes N=20,000, the power-law exponent γ=2.5, the minimum degree *k*_*min*_ = 2, and the structural cutoff $k_{c}=\sqrt{N}$ [37], where *c* is the normalization constant.

While the former network belongs to the group of networks with homogeneous degree distribution, the later is a network with power-law degree distribution, which is one of the common features shared by many real-world complex networks. Empirically, power law exponents in the range 2≲γ≲4 are common. Such a heterogeneous degree distribution tends to amplify the effect of degree-degree correlations on robustness and other properties of networks. Hereafter, we refer to these networks as Poisson and power-law networks, respectively.

For each setup, we generate 100 independent realizations of *G*_0_. From each *G*_0_, we apply Algorithm 1 with five values of *J*_1_ = −2*M*,−*M*,0,*M*,2*M* to generate 1-NNCRNs with varying *r*_1_, where *M* is the number of edges. Next, for each of these 1-NNCRNs, we apply Algorithm 2 with five values of *J*_2_ = −2*M*,−*M*,0,*M*,2*M* to control *r*_2_, resulting in 5×5=25 2-NNCRNs with distinct parameter sets $\left(J_{1},J_{2}\right)$ per initial *G*_0_, which means that for each $\left(J_{1},J_{2}\right)$ pair, we obtain 100 independently generated 2-NNCRNs.

Because each *G*_0_ is sampled from a random network ensemble, it does not contain structural correlations. Also, the irreducibility of both rewiring algorithms ensures that the equilibrium network ensemble only depends on the initial state through the degree sequence [31,32]. We use $10^{4}\times M$ rewiring steps to ensure convergence. Details on the convergence of *r*_2_ and the scalability of our algorithm are provided in Supporting Information.

### 2.3 Analyzing the structural robustness

The structural robustness of networks assesses the ease with which the global connectivity of the network is maintained. There are several strategies for destroying networks and processes of how networks fail [38–41]. Here, we investigate the most straightforward mechanisms, namely random node/edge failures, as well as targeted attacks removing nodes in decreasing order of degree.

As the fraction *p* of remaining nodes/edges decreases, the size of the largest connected component $N_{\mathrm{LCC}}(p)$ in the network decreases as well. For the network to maintain its functionality, global connectivity is necessary. Thus the value of *p* at which the largest connected component collapses, the critical point *p*_*c*_, serves as an essential indicator of robustness. We find it more intuitive to evaluate robustness using the fraction of removed nodes/edges that can be removed before collapse, $f_{c}=1-p_{c}$, so that a larger value of *f*_*c*_ indicates that the network is more robust.

In practice, $N_{\mathrm{LCC}}(p)$ does not always rapidly decrease around a single value of *p*. More precisely, the network may contain a densely connected core whose percolation occurs later than the more loosely connected periphery, which results in multiple phase transitions [42]. To illustrate this phenomenon, we compute the susceptibility

$$\chi (p)=\frac{\langle N_{\mathrm{LCC}}^{2}(p)\rangle -\langle N_{\mathrm{LCC}}(p)\rangle^{2}}{\langle N_{\mathrm{LCC}}(p)\rangle }.$$ (9)

The peak of the susceptibility indicates the location of critical points in a continuous phase transition and is often used to approximate *p*_*c*_. However, in situations as described above, the susceptibility exhibits multiple peaks, whose relative heights can change as the size of the network increases. These peaks can also merge or become indistinguishable, making it difficult to assign a well-defined critical point. This makes the peak position of susceptibility unsuitable for quantifying robustness. The multiple peaks phenomenon can be seen in the bottom right panel in Fig 4.

In this study, we instead approximate the critical point *p*_*c*_ using a threshold defined by

$$p_{1\%}=\sup\{p:\langle N_{\mathrm{LCC}}(p)\rangle <0.01N\},$$ (10)

where the largest component size falls below 1% of the total and use $f_{1\%}=1-p_{1\%}$ as a robustness measure. Our results are qualitatively unchanged for other small thresholds, as shown in Supporting Information. Note that if the threshold is set too large, the measure no longer captures the collapse point of the giant component. Instead, it begins to reflect the size of the giant component above the percolation critical point, which is captured by another robustness measure, *R*, introduced below.

Beyond the critical point, the size of the giant component as *p* varies also carries important information. Schneider *et al.* [23] proposed the area $R=\int_{0}^{1}S(p)\text{d}p$ under the curve $S(p)=N_{\mathrm{LCC}}(p)/N$ as a robustness measure. A larger value of *S*(*p*) indicates that the network is more robust for any fixed fraction *p* of remaining nodes/edges and *R* thus measures the average robustness away from *p*_*c*_.

In summary, network robustness can be interpreted using two measures: $f_{1\%}$ and *R*. A larger value of $f_{1\%}$ indicates that the network can maintain its global connectivity under a higher fraction of node/edge removals. The robustness measure *R*, defined as the area under the curve *S*(*p*), reflects the average connectivity across all node/edge removal scenarios, with a larger *R* indicating greater robustness.

To evaluate $f_{1\%}$ and *R* in a given network, we use the method proposed by Newman and Ziff [43].

In addition to structural robustness measures, we also compute the network efficiency *E*(*p*), defined as the average of the inverses of shortest path lengths between node pairs that remain connected after a fraction *p* of nodes or edges are removed [44]. Although *E*(*p*) does not directly measure connectivity, it reflects a functional aspect of information propagation or transport efficiency. We include this metric as it provides a preliminary indication that second-neighbor degree correlations (*r*_2_) have a non-trivial effect on functional robustness. Since this aspect is beyond the main scope of this study, we do not discuss the efficiency results in Sect 3, but refer to them briefly in Sect 5.2 as a direction for future work.

## 3 Result

In this section, we present the results of the numerical calculations based on the method described in Sect 2.2. First, we confirm that the algorithm works as desired, i.e., that the generated 2-NNCRNs exhibit various degree-degree correlations between both nearest-neighbor nodes and second-nearest-neighbor nodes. Next, we assess their robustness against random node/edge failures and degree-based targeted attacks.

### 3.1 Constraints on degree correlations in 2-NNCRNs

The purpose of this section is to give some details about the networks obtained by the rewiring procedure explained above, and in particular to explain how the parameters influence the range of correlations achievable with our method. More precisely, we confirm that degree distribution and *r*_1_ impose constraints to *r*_2_ and that the rewiring algorithm can sample network configurations with various *r*_2_ and the same *r*_1_.

Fig 2 depicts the average values of *r*_1_ and *r*_2_ of 2-NNCRNs generated by the rewiring method described in the previous section starting with an initial network characterized by (a) the Poisson degree distribution and (b) the power-law degree distribution. The confidence intervals defined as three times the standard error for the mean value of *r*_1_ and *r*_2_ corresponding to the different parameters $\left(J_{1},J_{2}\right)$ do not overlap, confirming that our sampling yields statistically distinct network sets. As shown in Fig 2, the range of *r*_2_ strongly depends on the degree distribution and *r*_1_. Let us comment on a few trends:

First, we note that *r*_2_ is shifted in the positive direction by larger absolute values of *r*_1_. Such positive extrinsic LRDCs have also been observed in NNCRNs in [7] and [45] and are consistent with the positive correlation of degrees between nodes at even distances. To understand this effect, note that if *r*_1_ is close to  + 1, similar degrees tend to be adjacent, leading to similar degrees at *l* = 2 and resulting in higher values of *r*_2_. On the other hand, if *r*_1_ is close to –1, high degrees tend to be adjacent to low degrees and vice versa. Due to this alternating pattern, we again find that nodes at distance *l* = 2 have similar degrees, which results in high values of *r*_2_.

Next, we observe that the range of *r*_2_ is narrower if the absolute value of *r*_1_ is large in the case of Poisson degree distributed networks (Fig 2(a)). For power-law networks, the converse is true but the effect is much weaker (Fig 2(b)). In other words, the range of *r*_2_ values achievable by 2-NNCRNs strongly depends on the value of *r*_1_ in Poisson networks, while the same trend is not observed for power-law networks. At present, we do not have a satisfying explanation for this behavior and we conclude that the relationship between *r*_1_ and *r*_2_ is complex and constrained by the degree distribution.

Finally, we mention that neither the range of *r*_1_ nor of *r*_2_ are symmetric. This is not surprising. For example, as explained above, both *r*_1_ close to  + 1 and *r*_1_ close to –1 tend to induce large positive values for *r*_2_, but the mechanism is different in both cases.

### 3.2 Random failure

Having confirmed our samples to be degree-degree correlated at distances *l* = 1 and *l* = 2 in various ways, we now investigate their robustness using the method described in Sect 2.3.

**Fig 3:**
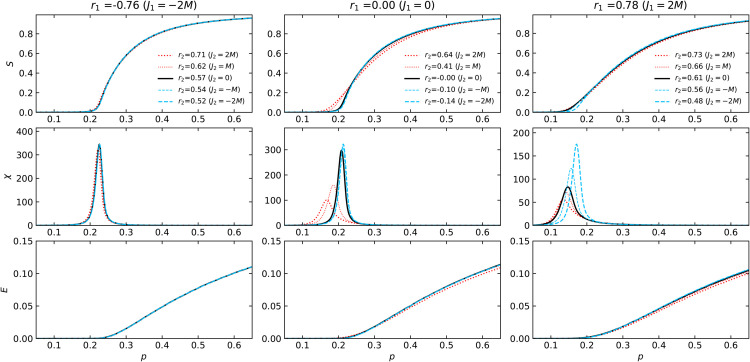
The S, χ, and E dependence on the edge retention probability p during random edge failure in Poisson networks. The set of 2-NNCRNs is the same as in Fig 2(a). The left, middle, and right panels correspond to rewiring parameters *J*_1_ = −2*M*, 0, and 2*M*, respectively. The colors and types of the lines correspond to rewiring parameters *J*_2_.

**Fig 4:**
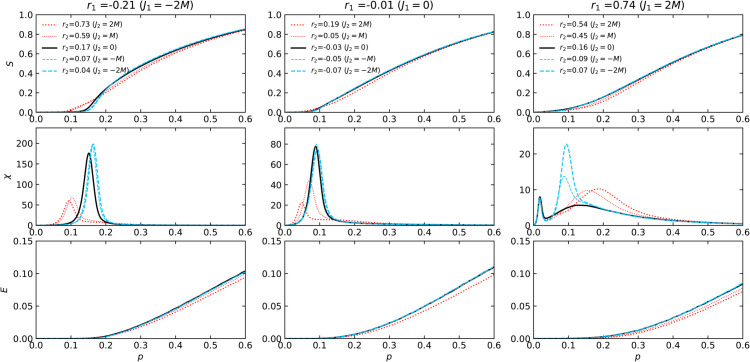
The S, χ, and E dependence on the edge retention probability p during random edge failure in power-law networks. Here we plot the same information as Fig 3 for the set of 2-NNCRNs from Fig 2(b).

Figs 3 and 4 depict the relative sizes of the largest connected component $S=\langle N_{\mathrm{LCC}}\rangle /N$ and the susceptibility χ (see Eq (9)) as functions of the probability *p* that an edge remains.

The solid black lines in Figs 3 and 4 represent the case of *J*_2_ = 0, i.e., the 1-NNCRNs without intrinsic correlations at distance l≥2, as the baseline for comparison. With the exception of the rightmost panel of Fig 4, the peak position of susceptibility suggests that *p*_*c*_ increases with larger *r*_2_-values, for fixed *r*_1_.

An exception to this trend is observed in power-law networks with a strongly assortative structure (*r*_1_ = 0.74), as shown in the bottom-right panel of Fig 4. As discussed in Sect 2.3, the susceptibility suggests the double-peak transition, which has been reported in networks with high clustering coefficients [42]. The double-peak transition is known to be characteristic of networks with a core-periphery structure. The peak at lower *p* appears consistently at *p* = 0.052 and is independent of *r*_2_, while the peak at larger *p* varies with *r*_2_. Notably, the direction of this variation is opposite to the trend seen in other cases, that is, larger *r*_2_ shifts the second peak toward smaller *p*.

Note moreover that for different values of *r*_2_ the relative order of *S*(*p*) flips at a certain crossing point, i.e., up to that point the giant connected component with large *r*_2_ is larger than the one corresponding to the network with small *r*_2_, while the reverse holds above the crossing point. In particular, for large value of *p* the giant component size grows more slowly and is smaller with larger *r*_2_. The two regions before and after the crossing point are aggregated into a single number *R*. Thus, when discussing the relationship between 2NNCRNs and robustness, it is necessary to look at both measurements, $f_{1\%}$ and *R*.

**Fig 5:**
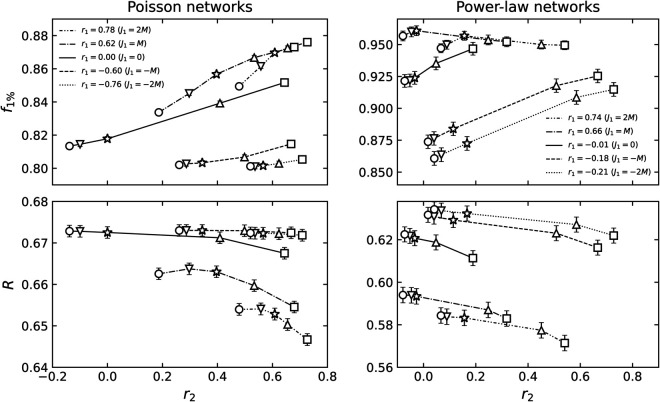
Robustness of 2-NNCRNs against random edge removal. The symbols indicate the parameter *J*_2_ and are the same as those in Fig 2, whereas lines connect points corresponding to 2-NNCRNs with the same *r*_1_ and the line type corresponds to *J*_1_. Each data point is the average value over 100 configurations, and the error bar is the standard deviation. The effect of *r*_2_ can be seen in the vertical spread of data points for a given line style, while the effect of *r*_1_ is seen in the vertical distance between different line styles. Note that these two effects are often comparable in magnitude.

The two measures $f_{1\%}$ and *R* used to characterize the robustness of 2-NNCRNs are derived from Figs 3 and 4 and the result is summarized in Fig 5. We observe that $f_{1\%}$ increases with *r*_2_ except for the case of assortative power-law networks, which means that the effect of perturbing *r*_2_ in the positive/negative direction away from $r_{2}^{\mathrm{ext}}$ is to make the network more robust/fragile. Conversely, *R* tends to decrease with increasing *r*_2_, which means that *r*_2_ has a negative effect on robustness quantified by *R*. Quantitatively, the size of the shift in the robustness measures resulting from a change in *r*_2_ is comparable to the effect of a change in *r*_1_, as explained in the caption of Fig 5. The shift induced by *r*_2_ is especially large in cases where the range of *r*_2_ is wide, such as Poisson networks with *r*_1_ = 0.00 or power-law networks with *r*_1_ = −0.74.

**Fig 6:**
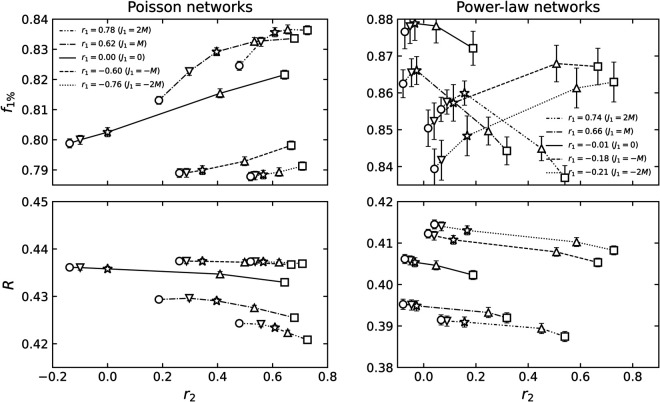
Robustness of 2-NNCRNs against random node removal. The line types and symbols are analogous those in to Fig 5. The stronger decrease in $f_{1\%}$ of assortative networks with *r*_1_>0 in the top-right panel, compared to edge failure, is likely due to the reduced accuracy of $p_{1\%}$ as an estimator of *p*_*c*_, especially when *p*_*c*_ is close to zero.

We have also performed the same analysis for random node failure, instead of edge failure. The results are similar to edge failure and are summarized in Fig 6.

### 3.3 Targeted attack

It is known that networks that are robust against random failures can still be vulnerable to targeted attacks, where nodes are removed in order of decreasing degree, in particular in random networks with power-law degree distribution [39,40]. On the other hand, when robustness is evaluated by *f*_*c*_, it is possible to be robust against both random failure and targeted attacks in networks with positive *r*_1_ [22]. Here, we investigate how the *r*_2_-dependence of robustness against targeted attacks differs from the case of random failure.

**Fig 7:**
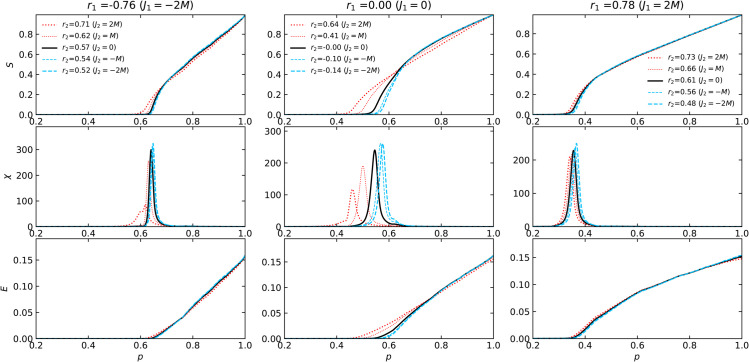
The S, χ, and E dependence on the node retention probability p during targeted attack on Poisson networks. This plot replicates the information from Fig 3 (Poisson networks) for the case of targeted attack.

**Fig 8:**
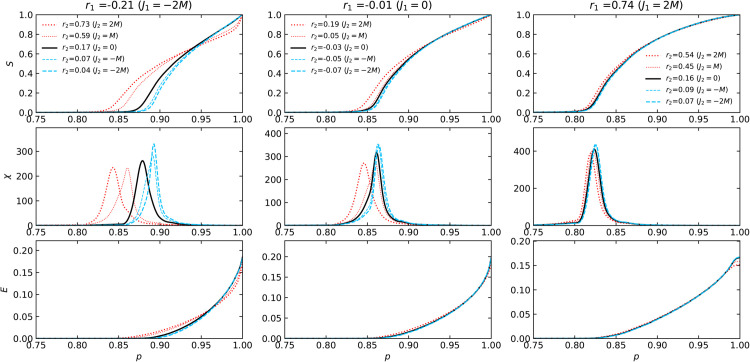
The S, χ, and E dependence on the node retention probability p during targeted attack on power-law networks. This plot replicates the information from Fig 4 (power-law networks) for the case of targeted attack.

**Fig 9:**
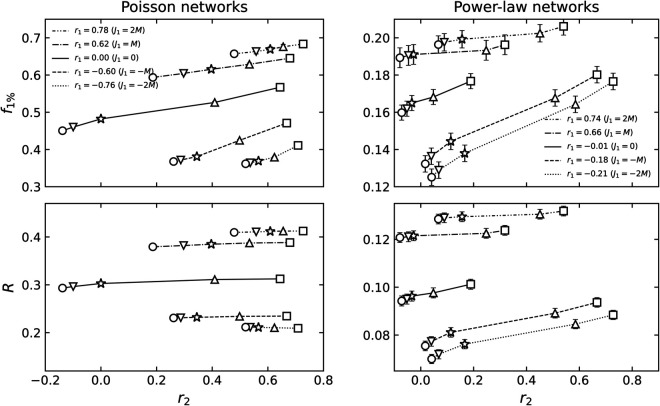
Robustness of 2-NNCRNs against targeted attack. The line types and symbols are analogous those in to Fig 5.

Figs 7 and 8 depict how the giant component size *S* and the susceptibility χ depend on the fraction *p* of surviving nodes under a targeted attack on the same 2-NNCRNs used in Figs 3 and 4. Fig 9 contains the corresponding values of $f_{1\%}$ and *R*.

We observe that $f_{1\%}$ increases with *r*_2_ for a fixed choice of *r*_1_ and that this behavior qualitatively matches our observation in the case of random edge failure. Quantitatively, the strength of the shift in $f_{1\%}$ in response to a change in *r*_2_ is stronger than in the case of random failure. Moreover, as in the case of random failure, the size of the shift in response to a change in *r*_2_ is somewhat weaker compared to a change in *r*_1_.

On the other hand, as observed from the bottom panels in Fig 9, there is a slight increase in *R* with respect to *r*_2_, which is more pronounced in power-law networks. We note that the direction of change in *R* in response is opposite to what we observed in random failure. This difference in behavior is due to the fact that, while there is still a crossing point between *S*(*p*) similar to the discussion in Sect 3.2, it occurs at a higher proportion *S*(*p*) of surviving nodes than in the case of random failure. This shift in the crosspoint occurs because targeted attacks rapidly destroy the network’s core, meaning any significant giant component only exists in a state of high fragmentation. In contrast, under random failure, the network degrades more smoothly, allowing the crosspoint to occur at a lower fraction of surviving nodes. Thus the values of *R* are influenced more by the behavior of the curves around *p*_*c*_ and we expect that the change in *R* more closely matches the change in $f_{1\%}$.

## 4 Conclusion

In this study, we have conducted numerical investigations into the structural properties of networks exhibiting degree-degree correlations between second-nearest neighbors, i.e., long-range degree correlations (LRDCs) at shortest path distance *l* = 2.

To achieve this, we have introduced *l*-th nearest-neighbor correlated random networks (*l*-NNCRNs), which exhibit degree-degree correlations up to the *l*-th nearest neighbor scale while being maximally random at any further scale. We have generated 2-NNCRNs using a two-step algorithm based on random edge rewiring and the Metropolis-Hastings algorithm for two representative degree sequences: Poisson and power-law distributions. Quantifying the strength of the LRDC at distance *l* using Pearson’s correlation coefficient *r*_*l*_, this two-step algorithm generates 2-NNCRNs with identical *r*_1_ but varying *r*_2_. Finally, we have investigated the robustness of the networks by simulating random node/edge failures and degree-based targeted attacks.

We have observed that the robustness quantified by $f_{1\%}$ is an increasing function of *r*_2_ when *r*_1_ is held constant in the almost all cases we simulated (Figs 5, 6 and 9). Thus, networks with smaller (larger) *r*_2_ than $r_{2}^{\mathrm{ext}}$ have been observed to be more fragile (more robust) against both random failure and targeted attack. On the other hand, the effect of *r*_2_ on network robustness, when measured by *R*, is more nuanced. In the case of bond percolation (Fig 5), *R* decreases as *r*_2_ exceeds $r_{2}^{\mathrm{ext}}$, indicating that the network becomes more fragile. However, in the case of targeted attacks (Fig 9), *R* remains constant or increases slightly with *r*_2_. This contrasts with our findings when robustness is quantified by $f_{1\%}$ and can be explained by the crosspoint-effect mentioned in Sect 3.2.

To summarize, our numerical results suggest that network robustness is correlated with *r*_2_ when *r*_1_ is fixed, as shown in Table 1. The trends in the influence of *r*_2_ on robustness are similar to those of *r*_1_. As seen in Figs 5, 6, and 9, the magnitude of the effect caused by changes in *r*_2_ is roughly half of that caused by *r*_1_, except in the case of *R* during targeted attacks. This highlights the significant role of second-neighbor degree-degree correlations in network resilience.

**Table 1:** Summary of effects of an increase in either *r*_1_ or *r*_2_ on $p_{1\%}$, $f_{1\%}$, and *R.*

	$p_{1\%}$	$f_{1\%}$	*R*
Random node/edge failure	↓*	↑*	↓
Degree-based target attack	↓	↑	↑

*This trend is inverted for assortative power-law networks, see Sect 5.1.

**Fig 10:**
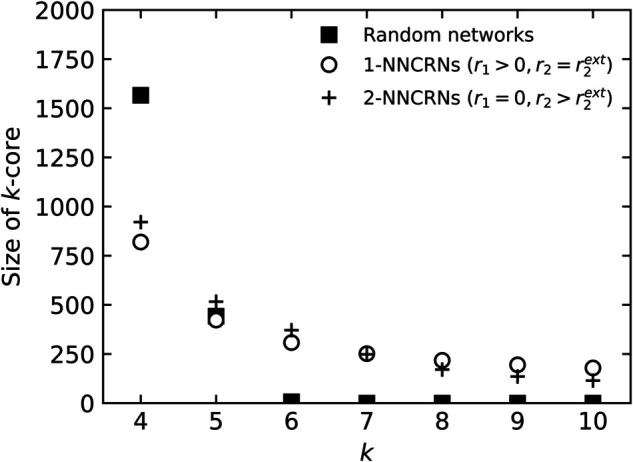
Size of k-cores in power-law networks with different degree correlations. The values are averaged over 100 configurations. Symbols represent different rewiring parameters: solid squares for $\left(J_{1},J_{2})=(0,0\right)$, open spheres for $\left(J_{1},J_{2})=(2M,0\right)$, and crosses for $\left(J_{1},J_{2})=(0,2M\right)$, which means that the networks are random networks, 1-NNCRNs, and 2-NNCRNs, respectively.

In conclusion, if *r*_1_ is kept fixed, then *r*_2_ has a similar effect on robustness as *r*_1_. To give some explanation of this effect, we have plotted in Fig 10 the size of the *k*-core [46] in power-law networks with strongly positive *r*_1_ or *r*_2_ and we observe that both constraints lead to quite similar values. To interpret this, recall that the *k*-core is the part of the network where the giant component starts to emerge as *p* approaches *p*_*c*_ from below and is thus intimately related to the robustness of the network. Quantitatively, the effect of *r*_2_ on robustness is smaller than *r*_1_ but comparable.

A simple, intuitive picture for why the effects of *r*_2_ on network robustness are so similar to those of *r*_1_ can be formed by imagining that each path of length two in the original network is replaced by a single “superedge”. The nearest-neighbor correlation *r*_1_ of this new superedge network would then correspond to the second-neighbor correlation *r*_2_ of the original network. This analogy by itself cannot amount to a complete explanation, as the state of two superedges originating from the same node is not independent (they may share their first edge in the original graph). Although not a formal proof, this conceptual model strongly suggests that *r*_2_ plays a structural role analogous to that of *r*_1_ in determining network robustness.

## 5 Discussion

While our main results reveal that *r*_2_ affects robustness qualitatively similar to *r*_1_, they also point to several nuanced phenomena. In this section, we examine an exceptional behavior observed in assortative power-law networks, where the robustness measure $f_{1\%}$ shows an unexpected trend and double-peak transitions. Afterwards, we discuss the implications of our findings, including potential applications to real-world network design, and outline directions for future research involving long-range correlations and higher-order network structure.

### 5.1 Exceptional behavior in assortative power-law networks

Here, we discuss an exceptional trend in $f_{1\%}$ observed in assortative power-law networks under random failures, see Sect 3.2 . This behavior can be attributed to the fact that the theoretical percolation threshold *p*_*c*_ is close to zero in this regime. In such cases, $p_{x\%}$ defined by any fixed positive threshold *x* no longer approximates *p*_*c*_, but rather reflects how quickly the giant component grows after *p* exceeds *p*_*c*_. For uncorrelated random networks, the theoretical threshold is given by the Molloy–Reed criterion $p_{c}=\langle k\rangle /(\langle k^{2}\rangle \;-\;\langle k\rangle )$, which equals 0.07 for the power-law networks we treat here, but assortative correlations may push the threshold even closer to zero. This makes $f_{1\%}$ behave differently from its usual interpretation as an indicator of connectivity collapse.

**Fig 11:**
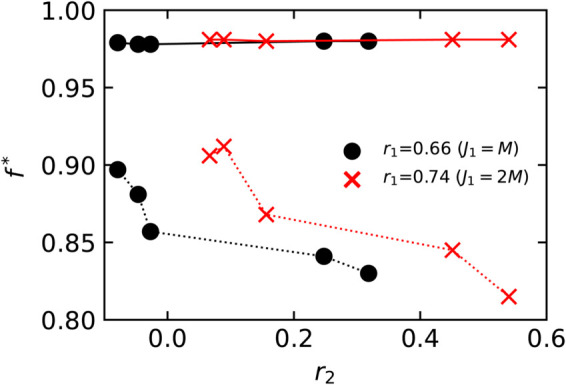
Robustness-like quantity f^*^ evaluated by the position of the peaks of susceptibility under random edge removal for the core (upper lines) and the periphery (lower lines). The set of networks is the same as the cases *r*_1_ = 0.74 and 0.66 at the right top panel of Fig 5).

Analyzing double-peak transitions helps us to understand this phenomenon. We extract the position p^*^ of both peaks and define a robustness-like quantity $f^{*}=1\;-\;p^{*}$ for each. Fig 11 shows resulting f^*^ values for both the first and second peaks as a function of *r*_2_. Although the largest f^*^ corresponding to the connectivity of the core remains stable under changes in *r*_2_, the smaller f^*^ corresponding to the periphery decreases as *r*_2_ increases. This observation is consistent with the fact that $f_{1\%}$ also decreases with increasing *r*_2_ in this regime, supporting the interpretation that both $f_{1\%}$ and the smaller f^*^ reflect the connectivity of the peripheral region rather than that of the core.

Interestingly, the enhancement of the second peak as $\left|r_{2}-r_{2}^{ext}\right|$ increases suggests a phenomenon specific to *r*_2_ that is not observed for *r*_1_. While *r*_2_ has been observed to have qualitatively similar effects to those of *r*_1_ throughout most of this study, this result highlights the distinct behavior of *r*_2_ in the context of double-peak transitions. These findings open new directions for future research on how long-range degree correlations influence structural phase transitions in networks.

### 5.2 Implications and further directions

**Fig 12:**
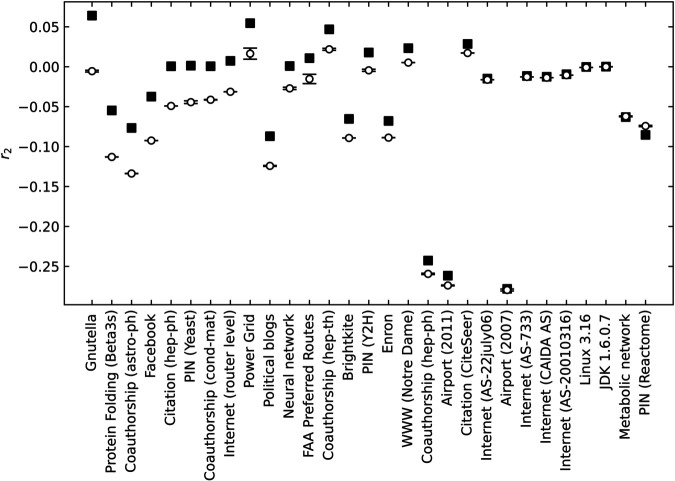
LRDCs at *l* = 2 in real networks in Table 2. Solid squares represent the *r*_2_-values for real-world networks. Open circles denote $r_{2}^{\mathrm{ext}}$ calculated as the average *r*_2_ values over 100 realizations of the corresponding 1-NNCRNs, with error bars indicating the standard deviation.

It is natural to expect that LRDCs at *l* = 2 also play an important role in the robustness of a real-world network. To investigate this, we considered 29 real-world networks from the dataset previously analyzed in [7], which were selected to represent a diverse range of systems under constraints such as the need for domain diversity, data accessibility, and computational feasibility. For these networks, Fig 12 summarizes the observed values of *r*_2_ as well as the value of $r_{2}^{\mathrm{ext}}$ for a 1-NNCRN with the same $P(k,k^{\prime }|l=1)$. In our dataset, most of the real-world networks exhibit larger *r*_2_ than $r_{2}^{\mathrm{ext}}$, and thus our results suggest that intrinsic LRDCs at *l* = 2 contribute positively to their robustness. Our result also suggests the applicability of *r*_2_ to control the robustness of real-world networks by changing *r*_2_ from $r_{2}^{\mathrm{ext}}$, which we will try to implement in future work. Note that the 2-NNCRN model discussed here is an idealization in the sense that we compare networks that only differ in their degree correlations, which will not necessarily be the case in real-world networks. Although our findings suggest the potential role of *r*_2_ in enhancing robustness, whether and how this effect can be harnessed or observed in real-world systems remains to be verified. It remains an important problem for future work to quantify the influence of LRDCs in such networks.

**Table 2:** Properties of the real networks in Fig 12.

Network	N	M	Ref.
Airport (2007)	500	2,980	[47,48]
Airport (2011)	1,574	17,215	[49]
Brightkite	58,228	214,078	[50,51]
Citation (CiteSeer)	384,054	1,736,145	[52,53]
Citation (hep-ph)	34,546	420,877	[50,54,55]
Coauthorship (astro-ph)	18,771	198,050	[50,56]
Coauthorship (cond-mat)	23,133	93,439	[50,56]
Coauthorship (hep-ph)	12,006	118,489	[50,56]
Coauthorship (hep-th)	9,875	25,973	[50,56]
Enron	36,692	183,831	[50,57,58]
FAA Preferred Routes	1,226	2,408	[52,59]
Facebook	63,731	817,035	[60]
Gnutella	10,876	39,994	[50,56,61]
Internet (AS-20010316)	10,515	21,455	[62]
Internet (AS-22july06)	22,963	48,436	[63]
Internet (AS-733)	6,474	12,572	[50,54]
Internet (CAIDA AS)	26,475	53,381	[50,54]
Internet (router level)	192,244	609,066	[64]
JDK 1.6.0.7	6,434	53,658	[52,65]
Linux 3.16	30,834	213,217	[52,65]
Metabolic network	453	2,025	[66,67]
Neural network	297	2,148	[63,68,69]
PIN (Reactome)	6,229	146,160	[52,70]
PIN (Y2H)	3,023	6,149	[52,71]
PIN (Yeast)	2,284	6,646	[72,73]
Political blogs	1,224	16,715	[63,74]
Power Grid	4,941	6,594	[63,69]
Protein Folding (Beta3s)	132,167	228,967	[62,75]
WWW (Notre Dame)	325,729	1,090,108	[50,76]

As mentioned in Sect 1, in addition to the effect of LRDC on structural robustness, it is natural to wonder about its effect on functional aspects of robustness. As a preliminary step in this direction, we have analyzed the behavior of the network efficiency *E*(*p*) which is plotted in the bottom rows of Figs 3, 4, 8 and 9. The *r*_2_-dependent trends in *E*(*p*) largely mirror those of the largest component size *S*(*p*), though they emerge at different stages: *S*(*p*) shows *r*_2_-sensitivity near the critical threshold, while *E*(*p*) exhibits variation even at high *p*, where the network remains largely intact.

**Fig 13:**
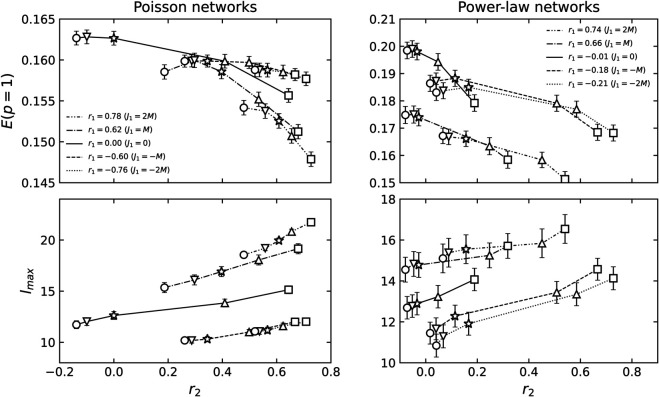
Efficiency and diameter of 2-NNCRNs. The line types and symbols are analogous to those in Fig 5.

To better understand this, Fig 13 shows how E(p=1) and the network diameter $l_{\max}$ depend on *r*_1_ and *r*_2_ in the absence of node/edge removal. We observe that both positive and negative shifts in *r*_1_ or *r*_2_ reduce *E*, although the underlying mechanisms differ. For strongly positive values, the reduction is associated with diameter expansion, consistent with enhanced core-periphery structure. For strongly negative values, diameter decreases, but the distribution of path lengths sharpens and shifts, implying homogenization of distances. In both cases, *r*_2_ influences efficiency in ways similar to *r*_1_. From the perspective of information spreading, these findings suggest that strong degree correlations (either positive or negative) can limit functional efficiency even before any failure occurs. However, as can be seen from Figs 3, 4, 8 and 9, below the critical pint *p*_*c*_ positive *r*_2_ can help maintain higher efficiency in the network.

Furthermore, previous work [77] has shown that degree assortativity plays a key role in cascade onset and size. We observed a similarly nuanced impact in a model of cascading failures, hence we have decided that a detailed investigation into these functional aspects, while promising, is beyond the scope of the present study and leave it for future work.

The insights gained from our study have important implications for the construction of more robust social systems and the development of effective contact rules to mitigate the spread of infectious diseases. In this context, one might wonder whether the scenario of “targeted attack” is practically relevant, since it presumes complete knowledge of the network structure (e.g., the number of contacts of all individuals threatened by an infectious disease). However, recent results have shown that similar attack schemes based on much more limited, local information yield essentially the same phase transition for scale free networks [78]. This suggests that our findings for the idealized targeted attack are likely to be robust.

Our results show that *r*_2_ significantly affects network robustness, with its positive impact explained by the *k*-core structure induced when $r_{2}>r_{2}^{\mathrm{ext}}$. Given that nonzero *r*_1_ has emerged as a fundamental property of network structure, influencing not only network robustness but also various dynamic processes and functions, it is likely that *r*_2_ plays a similar role. Further investigations are needed to explore the influence of *r*_2_ on other dynamic processes.

Regarding future directions of research, it is natural to wonder whether correlations at distance *l* = 3 and larger can play a similar role than those at distance *l* = 2. Conceptually, we expect that the importance of these correlations is quite small because, in a small-world network, the diameter scales like log(N) and thus the correlations up to distance *l* encompass correlations between almost all nodes, even for very small values of *l*. Moreover, our approach based on network rewiring becomes challenging even in the case *l* = 3, since no irreducible method to choose two edges preserving second-nearest neighbor degree correlations is known. Moreover, note that in Algorithm 2 the most time-consuming step is to compute the updated value $r_{2}^{\mathrm{next}}$, which requires an update to all neighbors of $\left\{u_{1},u_{2},v_{1},v_{2}\right\}$ and can thus be done in time $𝒪(\langle k^{2}\rangle^{2}/\langle k\rangle^{2})$, see also Supporting Information. In the case *l* = 3, we need to consider nodes up to distance 2 from $\left\{u_{1},u_{2},v_{1},v_{2}\right\}$ and thus the time-complexity will be much higher (we expect it is be $\langle k^{2}\rangle^{3}/\langle k\rangle^{3}$, depending on the implementation of the local update).

## Supporting information

S1 Appendix Additional analyses and methodological details. Contains a detailed discussion of the scalability of the rewiring algorithm, other ensembles with long-range degree correlations as well as the effect of threshold value.
(PDF)
