# Supplementary material for: Structural robustness of networks with degree-degree correlations between second-nearest neighbors
Source: PLoS One. 2025 Dec 5;20(12):e0336970. doi: 10.1371/journal.pone.0336970 (PMC12680194; doi:10.1371/journal.pone.0336970)
Supplement: S1 Appendix — Contains a detailed discussion of the scalability of the rewiring algorithm, other ensembles with long-range degree correlations as well as the effect of threshold value. (PDF) [file pone.0336970.s001.pdf]

Structural robustness of networks with degree-degree correlations between second-nearest neighbors

Supporting information

Yuka Fujiki[1,2◑*], Stefan Junk[3◑]

1 Frontier Research Institute for Interdisciplinary Sciences (FRIS), Tohoku University, 6-3 Aramaki aza Aoba, Aoba-ku, Sendai 980-8578, Japan.
2 Advanced Institute for Materials Research (AIMR), Tohoku University, 2-1-1 Katahira, Aoba-ku, Sendai, 980-8577 Japan.
3 Gakushuin University, 1-5-1 Mejiro, Toshima-ku, Tokyo 171-8588 Japan.

◑These authors contributed equally to this work.
* yfujiki@tohoku.ac.jp

1 Scalability of rewiring algorithm

We measure the computational effort of Algorithm 2 by counting the number t_a of accepted rewiring steps. As shown in Fig 1, the value of r_2 stabilizes after a sufficiently large number of accepted rewiring steps, indicating that the system has reached equilibrium. Based on experiments for networks with varying sizes ($N = 2 \times 10^2$, 2×10^3, 2×10^4), we determined that the number of accepted steps required for equilibration equals $10^4 \times M$, where M denotes the number of edges. Furthermore, Fig 2 shows the acceptance rate t_a/t (the fraction of rewiring steps that are accepted) in equilibrium as a function of network size. The fact that this acceptance rate is bounded away from zero indicates that the algorithm efficiently explores the state space without getting trapped in locally optimal configurations.

In Algorithm 2, each rewiring step requires the computation of r_2, which depends on the joint degree distribution $P(k, k'|l = 2)$ between node pairs at distance two. Assuming that small cycles that causes overlapping second-nearest neighbors can be neglected, the number of distance-two node pairs can be approximated by $\sum_k N_k k(k_{\mathrm{nn}}(k) - 1)$, where N_k is the number of nodes with degree k, and $k_{\mathrm{nn}}(k)$ is the average degree of neighbors of k-degree nodes. For uncorrelated random networks, $k_{\mathrm{nn}}(k)$ is approximated by $\langle k^2\rangle/\langle k\rangle$, leading to a total number of distance-two node pairs proportional to $N\langle k(k-1)\rangle$. Hence, the naive cost of recomputing r_2 at each step would be $\mathcal{O}(N\langle k(k-1)\rangle)$.

However, in our implementation, we maintain and update $P(k, k'|l = 2)$ incrementally by modifying only the local neighborhoods of the four endpoints of the two edges being rewired. Due to the bias in edge selection (proportional to $kP(k)/\langle k\rangle$), the expected update cost per step can be estimated as proportional to $\langle k^2\rangle\langle k(k-1)\rangle/\langle k\rangle^2$ under the assumption of no degree correlations. Given an acceptance rate $\tau = t_a/t$, the total number of rewiring steps required to reach $t_a = 10^4 \times M$ accepted steps becomes $t = 10^4 \times M/\tau$. Therefore, the total computational cost of Algorithm 2 can be estimated as $\mathcal{O}\left(M\langle k^2\rangle\langle k(k-1)\rangle/\langle k\rangle^2\tau\right)$, and change depending on the degree sequence and the (J_1, J_2) parameter settings.

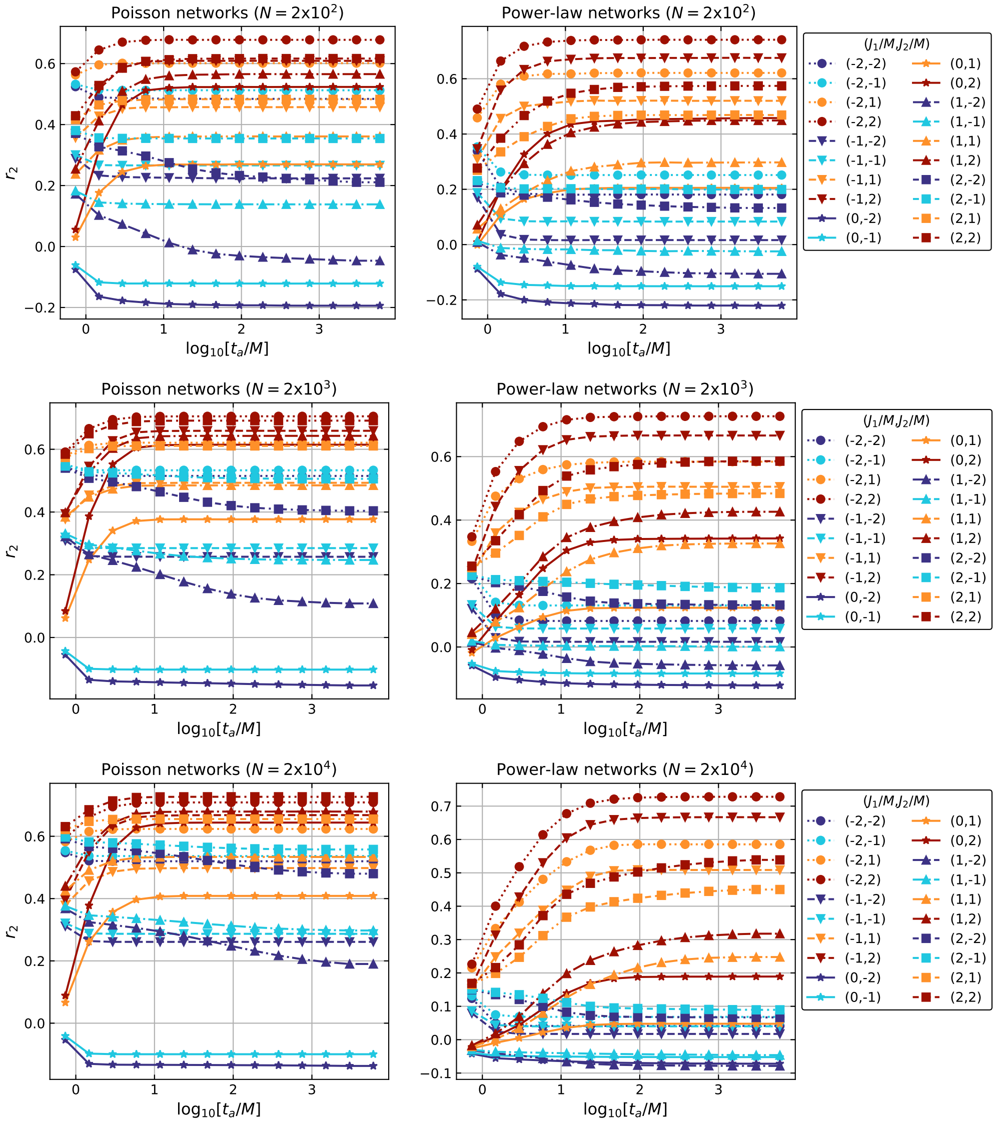

Fig 1. Convergence behavior of r_2 during the Algorithm 2. The horizontal axis shows the number of accepted rewiring steps t_a, and the vertical axis shows the corresponding value of r_2. Each line corresponds to a different rewiring parameter set (J_1, J_2).

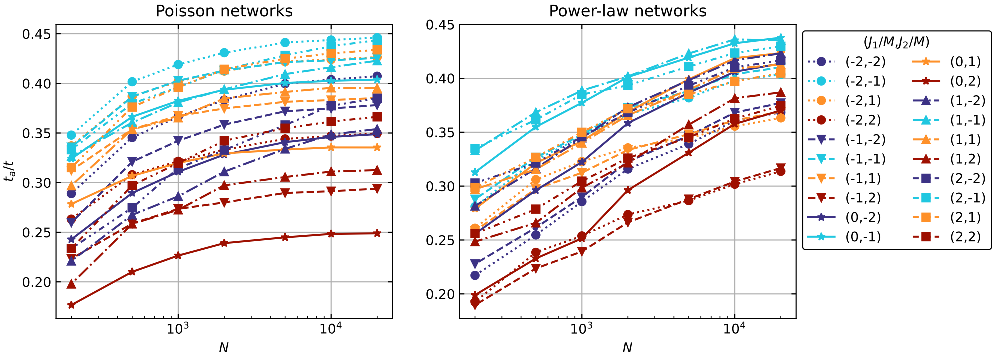

Fig 2. Acceptance ratio t_a/t of rewiring step during the Algorithm 2. The horizontal axis shows the number of nodes N, and the vertical axis shows the corresponding value of t_a/t. Each line corresponds to a different rewiring parameter set (J_1, J_2).

2 Other ensembles with long-range degree correlations

Let us briefly compare l-NNCRNs to other models with LRDCs.

Stochastic block model The stochastic block model is often used to represent networks with a known community structure. Here, nodes are partitioned into communities $C_1, \ldots, C_p$ of comparable sizes and one specifies the connection probabilities within each community and across communities (it is further possible to do this with a prescribed degree sequence). LRDCs occur if the average degrees d_i within each community C_i are different and not all communities are closely connected, so that nodes with degrees d_i and d_j from communities C_i and C_j typically connect through a third community C_k. Note that the effect of degree correlations can be analyzed using specific features of the model. For example, the critical point of the ensemble is the minimum of the critical points within each community.

Hierarchical modular networks Hierarchical modular networks generalize the idea of the SBM by presuming that the nodes are embedded in a hierarchy of modules, i.e., the nodes form the initial level and each successive level encodes the community structure among the elements of the previous level. The SBM thus captures the hierarchy up to the second level and a similar mechanism to before can explain LRDCs. While such structures appear to be quite common in real-world networks [1], their robustness is best analyzed by taking into account the details of the network under consideration.

Modular interacting network A variation of the previous model is the modular interacting network, where the community structure itself is randomized. This framework is best understood as a "network-of-networks", that is, a larger network whose nodes are themselves networks. Such a model inherently produces long-range degree correlations that depend on both the internal structure of the node-networks and the topology of their connections. As before, these systems are best analyzed using model-specific techniques that account for the two levels of structure, as has been done in [2].

dk-series Beyond the probabilities $P(k, k'|l)$ used in this work, it is also worth mentioning an alternative way of specifying degree correlations called the dk-series [3], which counts the number of motifs of up to k nodes together with their degrees. For example, the only motif with $k = 1$ (resp. $k = 2$) nodes are single nodes (resp. edges), so the $d1$ series (resp. $d2$ series) is just the degree sequence (resp. the matrix $P(k, k|l = 1)$). There are two motifs involving $k = 3$ nodes (triangles and paths) and prescribing the $d3$ series of a network becomes quite restrictive. Note that for two nodes at distance $l = 2$, each connection is counted towards the $d3$ series whereas the pair only contributes once to the matrix $P(k, k'|l = 2)$, so these methods are quite different.

3 Effect of threshold value

In Figs 3-4, we plot threshold values $f_{x\%} = 1 - p_{x\%}$, where $p_{x\%} = \inf\{p\colon S(p) > \frac{x}{100}N\}$ is the fraction of remaining nodes or edges needed for the largest connected component to reach x percent, for various values of x. It can be seen that all values have a very similar behavior and thus our particular choice does not bias the result.

References

1. Song C, Havlin S, Makse HA. Origins of fractality in the growth of complex networks. Nature Physics. 2006;2(4):275–281. doi:10.1038/nphys266.

2. Dong G, Wang F, Shekhtman LM, Danziger MM, Fan J, Du R, et al. Optimal resilience of modular interacting networks. Proceedings of the National Academy of Sciences. 2021;118(22). doi:10.1073/pnas.1922831118.

3. Orsini C, Dankulov MM, Colomer-de Simón P, Jamakovic A, Mahadevan P, Vahdat A, et al. Quantifying randomness in real networks. Nat Commun. 2015;6(1):8627. doi:10.1038/ncomms9627.

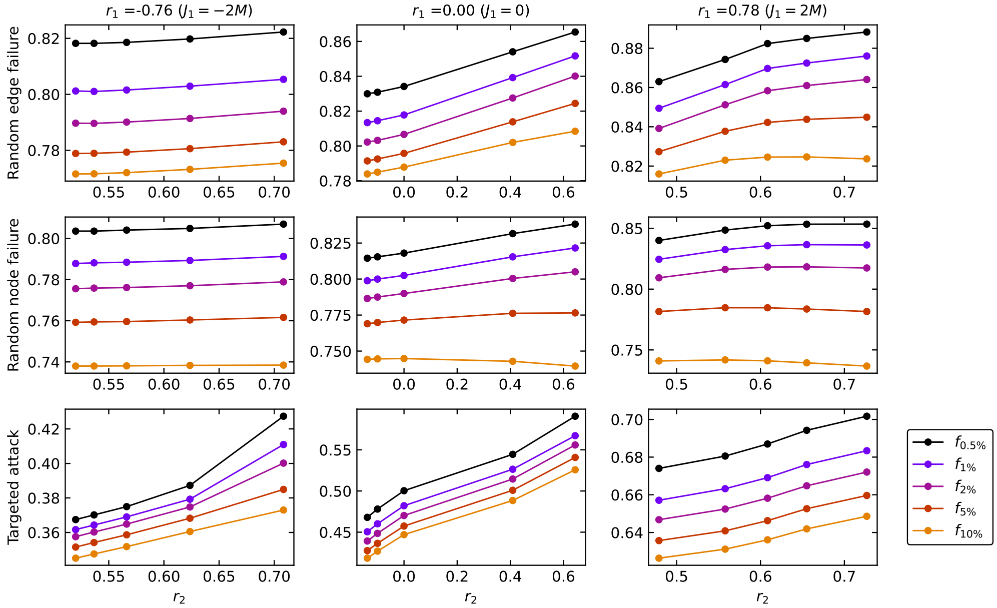

Fig 3. The threshold values $f_{x\%}$ for Poisson networks. The set of 2-NNCRNs is the same as Fig 1(a) of the main text. The top, middle, and bottom panels present the results for random edge failure, random node failure, and targeted attack, respectively. The left, middle, and right panels correspond to rewiring parameters $J_1 = -2M$,0, and $2M$, respectively.

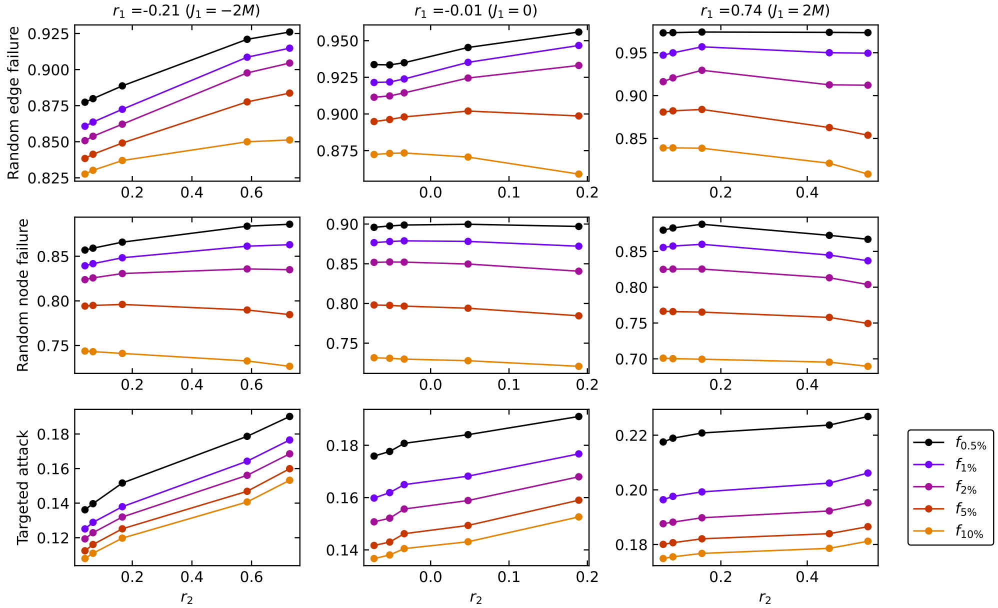

Fig 4. The threshold values $f_{x\%}$ for power-law networks. Here we plot the same information as Fig 3 for the set of 2-NNCRNs from Fig 1(b) in the main text.
